# Elastic Energy Storage in Biological Materials: Internal Stresses and Their Functionality

**DOI:** 10.1002/adma.202508442

**Published:** 2025-07-28

**Authors:** Shahrouz Amini, Paul Zaslansky, Boaz Pokroy, Peter Fratzl

**Affiliations:** ^1^ Department of Biomaterials Max Planck Institute of Colloids and Interfaces 14476 Potsdam Germany; ^2^ Max Planck Queensland Centre Brisbane QLD 4000 Australia; ^3^ Department for Operative Preventive and Pediatric Dentistry Charité – Universitätsmedizin 14197 Berlin Germany; ^4^ Department of Materials Science and Engineering and the Russell Berrie Nanotechnology Institute Technion−Israel Institute of Technology Haifa 320000 Israel

**Keywords:** active materials, elastic energy storage, internal stresses, kinematic amplification

## Abstract

In the biological world, materials are often heterogeneous and anisotropic, comprising components with very different elastic properties. The resulting structures are exposed to force generation by chemo‐mechanical energy conversion—such as water absorption, phase separation, or crystallization. Such phenomena may result in strain misfits that generate internal stresses that store elastic energies, which turn out to be extremely useful for enabling functions such as shape change, locomotion, or predation. However, the significance of elastic energy storage has received little attention. In this review, by considering examples of a broad spectrum of biological materials spanning shape‐morphing plant seed pods, smart appendages of crustaceans, ballistic tongues, and damage‐tolerant mineralized tissues, the fundamental aspects involved in the generation, storage, and release of internally generated elastic energies are surveyed. These have major implications for functions such as strengthening and toughening, shape morphing and actuation, ballistic movements, tensegrity, and bending stabilization. How Phenomena such as atomic or protein incorporation into minerals, conformational changes of proteins, phase transformation, and osmotic pressure are manipulated in the biological world to generate function by storing elastic energy are described. Such “elastic energy batteries” provide efficient performance and evolutionarily adapted functionality through a smart, structure‐based energy management.

## Graphical abstract

Harnessing and storing internally generated elastic energy is a clever strategy by biological materials to perform functions like shape transformation, movement, and predation. This review explores how biological systems manipulate mechanisms like atomic or protein integration into minerals, protein conformational shifts, phase transitions, and osmotic pressure to store and utilize elastic energy—functioning as “elastic energy batteries” to drive biological processes.

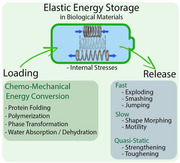

## 1 Introduction

Generating mechanical forces is central to initiating and controlling motion and functionality in many biological systems.^[^ ^1^ ^]^ Examples can be found in many organisms, for example the extension of the actin cytoskeleton for cell migration,^[^ ^2^ ^]^ muscle contraction and relaxation for locomotion^[^ ^3^ ^]^ or predation,^[^ ^4^, ^5^ ^]^ plant cell expansion for growth,^[^ ^6^ ^]^ opening seed pods for spreading pollen for reproduction,^[^ ^7^ ^]^ the accommodating internal stresses for toughening brittle biominerals^[^ ^8^ ^]^ and so forth. The energy required for performing such mechanical work is often supplied through a chemo‐mechanical conversion (whether using metabolic energy or by moisture or heat exchange). This energy can be directly used to perform the needed work, or it may become stored elastically to foster delayed functions. Notably, the harnessed energies generated by biofuels are often not sufficient for many essential biological functions, for example, reproduction using seed and pollen dispersal,^[^ ^7^ ^]^ defense and predation,^[^ ^5^ ^]^ and nutrition.^[^ ^9^ ^]^ Storage of elastic energy in nature takes on many sophisticated strategies to increase the power output, accelerating the release rate beyond the chemo‐physical limits, making it possible to enact delayed functions even when the biofuels are no longer available (e.g., post‐mature seed release). Therefore, in this review, we discuss phenomena involved in feeding nonmechanical energy into natural mechanical systems, storing it in the material, and thereafter releasing it, to realize functions driven by internal elastic energy.

**Figure 1:**
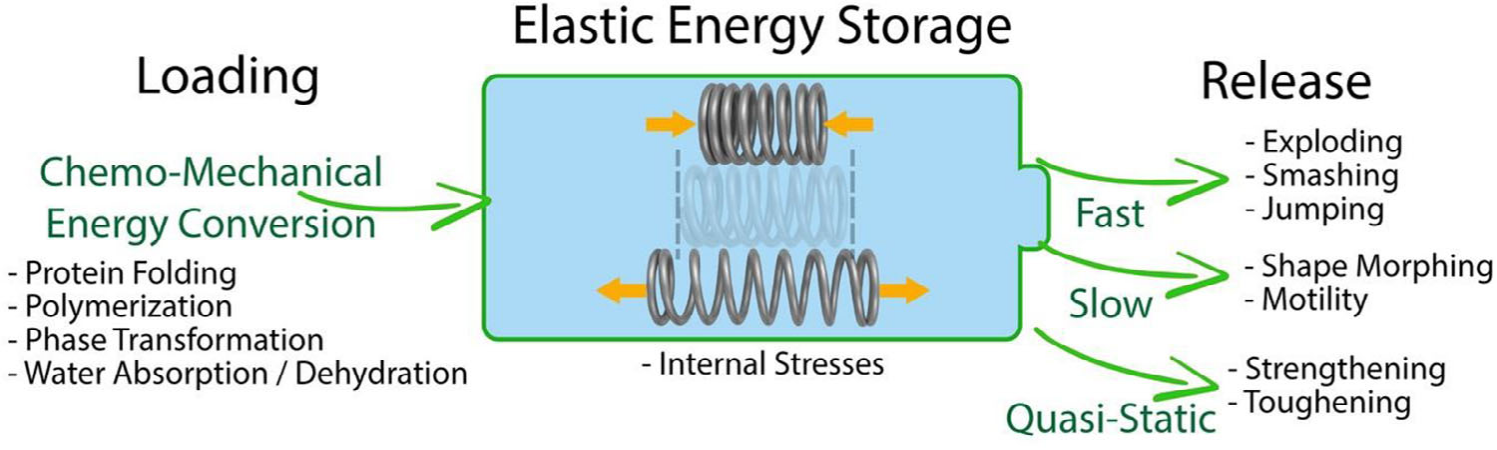
Schematic flow of elastic energy employed in many biological systems. Chemo‐mechanical energy conversion drives loading, limited by metabolic constraints. This can be stored as elastic energy and then released at a relevant rate, well‐matched with the desired biological functions.

The elastic energies in muscle‐driven movements,^[^ ^3^, ^10^, ^11^ ^]^ biomineralized tissues,^[^ ^8^ ^]^ and their benefits for pre‐stressed engineering materials^[^ ^12^ ^]^ have been covered in several reviews. Therefore, the present work primarily aims to provide an overview of the widespread occurrence of internally generated elastic energy storage and amplification across diverse species, with a focus on cases where temporarily stored elastic energy plays a major role in the function of biological materials. We provide examples of biological functions in which internal stresses are stored in the tissue, often after being generated through chemo‐mechanical conversion (**Figure** **1**). This stress is a source of energy that may enable a number of different biological functions, depending on its release rate, as graphically depicted in Figure 1. We thus introduce the primary mechanisms involved in the generation and storage of internal stresses, and we provide an overview of experimental methods employed to investigate internal strains in biological materials.

Storage of elastic energy, *U*, is a function of capacity that depends on the strained volume, the elastic modulus (*E*), and the strain (ɛ). As a result, soft tissues require high strains to generate substantial elastic energy, whereas materials with high elastic modulus, such as mineralized tissues, only require subtle strains to generate vast elastic energies. Such energy and consequent internally generated stresses can be used in both tension and compression. Depending on the material's inherent properties, tensile stresses are often stored in a polymeric matrix, for example, resilin (in arthropods), cellulose (in plants), collagen (e.g., tendon), or elastin (in mammals). In contrast, the stiff ceramic phase contained in mineralized tissues, such as teeth, bone, and mollusk shells, is well capable of storing compressive elastic energy. In general, elastic equilibrium requires that tensile stresses are always compensated by compressive stresses in other parts of the tissue, which is why hybrid materials (where one component resists compression and the other tension) are ideal for storing elastic energy.

Internal stresses typically result from eigenstrains, which are often the result of a physicochemical process, such as the absorption of water (that locally increases the volume), conformational changes in molecules (that modify length), or the inclusion of defects during crystallization (creating strains in the crystal lattice). With the appearance of eigenstrains, internal elastic stresses develop, establishing a mechanical equilibrium in the material. In industrial processes, internal stresses (such as in metallic engineering materials) are often introduced by large deformations, so that plastic deformation results as an eigenstrain that is compensated by (residual) elastic stresses.^[^ ^13^ ^]^ This, however, is rarely observed in biological materials that are most often polymers, minerals, or a combination of both. And yet, the physicochemical processes mentioned above are just as effective in introducing internal stresses in the biological world.

## 2 Functions

In the following, we briefly outline the biological functions supported by the storage and release of elastic energies, which we sort based on energy release rate as introduced in Figure 1, grouped into quasi‐static, slow and fast release, as addressed in Section heads “Strengthening and Toughening”, “Shape‐Morphing Actuation”, and “Elastic Energy Storage for Rapid Movements”, respectively.

### 2.1 Strengthening and Toughening

**Figure 2:**
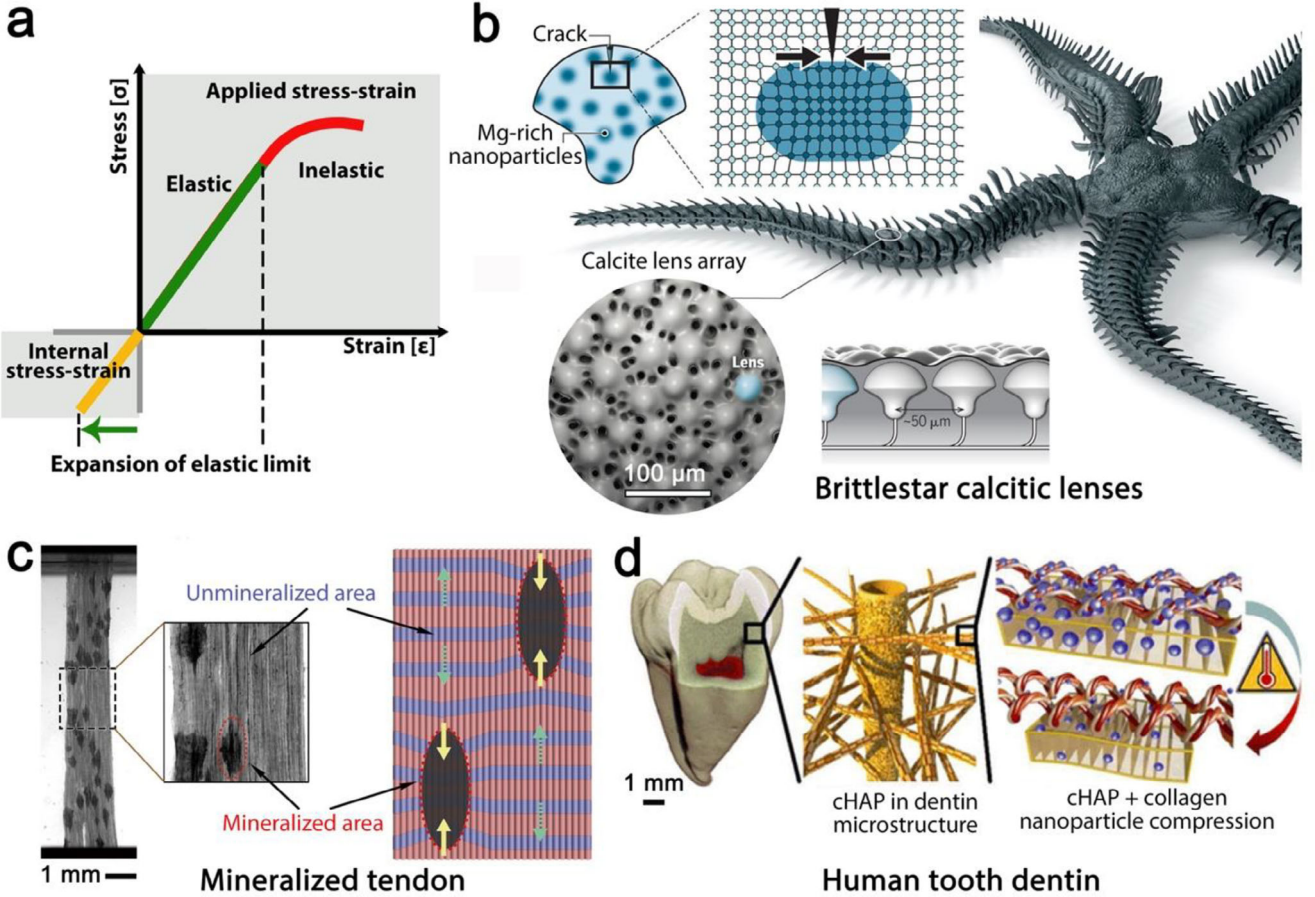
Storage of elastic energies in mineralized tissues promotes the strengthening and toughness of the structure. a) Internal stresses expand the range where the material can respond elastically by promoting energy dissipation (the area under the stress‐strain curve). This strategy has been adapted by mineralized tissues such as b) brittlestar calcitic lens, c) mineralized tendon, and d) human tooth dentin to enhance mechanical performance. Panels b, c, and d are adapted with permissions from^[^ ^34^ ^]^ Copyright 2017, AAAS,^[^ ^26^ ^]^ Copyright 2022, AAAS, and^[^ ^28^ ^]^ Copyright 2016, American Chemical Society, respectively.

Strength, the stress that a material can withstand prior to experiencing failure and toughness, the energy that a material can absorb before failure, are key to the mechanical performance of materials. When internal and applied stresses operate in opposite directions, so that the applied stress is partially compensated, both strength and toughness of the material can benefit from the presence of internal stresses. The way in which internal stresses affect these two material characteristics relies on different phenomena: in the case of strength, the presence of internal stresses can expand the response range in which a material behaves elastically (**Figure** **2**a), so that internal stresses allow further accommodation of elastic deformations. This is well‐known in engineering, where internal stresses can improve strength, for example, in tempered glass.^[^ ^14^ ^]^ Moreover, internal stresses alter the stress distribution resulting from externally applied forces so that elastic energy may be released through nano‐/micro‐cracking and plasticity rather than brittle fractures.

Remarkably, biological materials can leverage both scenarios–enhancement of strength and toughness– to improve their mechanical properties and surpass the mechanical limits of their base materials. For example, biomineralized tissues such as teeth, bones, and mollusk shells, which are primarily employed for mechanically demanding functions such as support, mastication, and protection, are mainly composed of brittle ceramics that withstand substantial compressive forces but tend to be weak under tension forces. Nature addresses this weakness by artfully utilizing compressive internal stresses that can be used to enhance the load‐bearing capacity.^[^ ^8^, ^15^, ^16^ ^]^ This is achieved by implementing three different approaches: i) incorporation of organic inclusions in the mineralized matrix, ii) inorganic inclusions (often anisotropically distributed) by means of localized chemical misfits, and iii) matrix‐induced prestresses.

- Mineralized tissues, also known as biological ceramics, unlike most of their engineered counterparts, are crafted through an organic‐mediated deposition process known as biomineralization.^[^ ^17^, ^18^ ^]^ Stepwise formation and highly controlled growth lead to the development of hierarchical structures with superior properties. Organic‐mediated development comes with the need to create interfaces between the organic and inorganic building blocks. This introduces heterogeneities with potentially incompatible properties, which may incorporate a substantial portion of organic inclusions in the mineralized matrix.^[^ ^8^ ^]^ Mechanical mismatches and structural “imperfections” can originate and induce internal stresses within biological ceramics. In mollusk shells,^[^ ^19^, ^20^ ^]^ corals,^[^ ^21^ ^]^ and coccolith,^[^ ^22^ ^]^ which are mainly composed of calcium carbonate crystals, the presence of a small amount of intracrystalline organic inclusions induces anisotropic lattice distortions. These generate local strain fields in the host crystals. The internal stresses increase the hardness^[^ ^23^ ^]^ and toughness,^[^ ^24^ ^]^ thus enhancing the properties of otherwise inherently brittle structures.
- In the calcitic microlenses of the brittlestar, the segregation of magnesium‐rich nanoparticles locally forms compressive stresses in the crystalline host matrix. This is attributed to the substitution of calcium with magnesium, which, due to the smaller atomic radius of Mg, results in a shrinkage of the crystalline lattice spacing. Thus, Mg‐rich nanodomains, which form during or just after amorphous to crystalline transformation, exert compressive strains on the matrix while sustaining high tensile strains^[^ ^25^ ^]^ (Figure 2b). In the specific case of calcite, the induced lattice mismatch generates ≈170 MPa compressive stresses. This substantially impacts the probability of crack formation since crack development would require tension stresses that must overcome the elastic energy barriers of the entrapped compressive stresses. This phenomenon allows the toughness to increase twofold. Notably, these internally generated stresses can be partially removed by annealing, which induces Mg diffusion in the lattice.^[^ ^25^ ^]^
- In tissues with a mineralized collagenous matrix, such as those found in mineralized turkey tendon,^[^ ^26^ ^]^ human tooth dentin,^[^ ^27^, ^28^ ^]^ and mammalian and fish bones,^[^ ^16^, ^29^, ^30^, ^31^ ^]^ internal stresses can reach values as high as several megapascals.^[^ ^32^ ^]^ The generation of internal stresses has been attributed to mineral precipitation in and around the collagen fibrils, where mineral replaces water in the collagenous matrix.^[^ ^16^ ^]^ The dehydration results in a conformational change of the collagen triple helix^[^ ^33^ ^]^ and shortening of the collagen fibrils, which consequently generates large contractile forces within the collagen matrix and induces compressive forces on the mineral domains (Figure 2c,d). Accordingly, internal compressive forces play a role in enhancing the strength of these mineralized tissues.^[^ ^16^ ^]^

### 2.2 Shape‐Morphing Actuation

**Figure 3:**
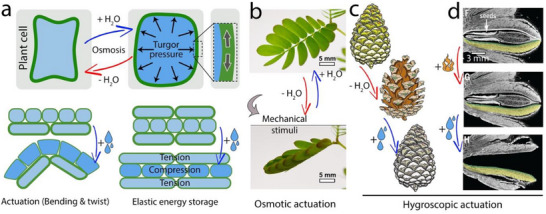
Examples of shape‐morphing and actuation in plant tissues. a) While the real‐time relaxation of generated mechanical energy can be used to promote material motility, accumulation of elastic energy and the buildup of stress can promote mechanical instability that eventually becomes the source of high‐energy movements and fast energy release, such as those required for ballistic seed dispersal (see Section head "Elastic Energy Storage for Rapid Movements"). b) Internal stresses in cell walls regulated by cell turgidity form reversible hydraulic actuation in mimosa leaves following a poroelastic timescale, which is controlled by plant cell osmolarity. The schematic image is adapted with permission from^[^ ^42^ ^]^ Copyright 2016, AAAS c) Reversible hygroscopic actuation in pine cones relies on a mismatched swelling/shrinkage and generation of elastic energy in the bi‐layer scales. d) Banksia seedpods take advantage of stored elastic energies to trigger stepwise irreversible actuation for delayed seed release. The images are adapted with permission from^[^ ^7^ ^]^ Copyright 2017, Wiley.

Unmineralized biological materials, such as plants and muscles containing cell‐level actuators, are capable of accommodating large mechanical strains simply because of their lower elastic modulus. Such large induced internal strains can lead to movements, actuations, and morphological transformations. In plants, motility is often generated by i) osmotic‐based actuation, which directly impacts the cell volume and length and relies on internal metabolic energies in living cells, or by ii) hygroscopic actuation based on the diffusion of water molecules caused by humidity differences between the tissue and atmosphere.^[^ ^6^ ^]^ This means that actuation results from internal stresses generated by the incompatible elasticity of an isotropically swelling hydrogel and anisotropic geometrical constraints created by stiff fibers or cell walls^[^ ^35^, ^36^, ^37^ ^]^ (**Figure** **3**a). These internal stresses may be released immediately, at least in part, through geometrical transformation (e.g., bending, twisting), and/or they may accumulate and become stored in the tissue for delayed relaxation upon a further stimulus.

An example of osmolarity‐driven actuation can be found in mimosa leaves, where chemo‐mechanical conversion occurs through changes in salt concentration in the vacuoles of Pulvinus endodermal cells, which act as motor organs.^[^ ^38^ ^]^ Accordingly, increased intracellular salinity results in a hypotonic environment and cell turgidity. In contrast, moving salt ions out of cells decreases the turgid pressure, resulting in a hypertonic environment and flaccidity (Figure 3b). This reversible motion follows the poroelastic timescale defined by tissue hydraulic characteristics^[^ ^39^ ^]^; thus, real‐time shape‐morphing actuation releases the main fraction of the internally generated stresses. In addition to actuation using metabolic energy, actuation using ambient moisture and temperature can drive the diffusion of water molecules caused by the humidity difference in the tissue. Consequently, the atmosphere can drive reversible or irreversible movements such as those found in non‐living tissues in pine cones and wheat awns,^[^ ^35^, ^40^ ^]^ Bauhinia variegata^[^ ^37^ ^]^ (orchid tree), and Banksia seedpods.^[^ ^7^, ^41^ ^]^

The manner in which a pine cone opens (and closes) is a good example of reversible hygroscopic actuation in which the scales open upon dehydration and close upon rehydration.^[^ ^35^, ^40^ ^]^ Anisotropic deformation is induced in the scale due to differently structured and strongly connected layers with internally generated stresses; the confinement of humidity‐driven actuation along rigid cellulose microfibrils at the inner scale walls results in minor strains and elastic energy storage; in contrast, unconfined polymeric matrix at the outer wall of the scales support stress relaxation that results in a pronounced shrinkage and swelling (Figure 3c). In a similar manner, the seedpod of *Bauhinia variegata* (orchid tree) exhibits a bilayer‐inducing hygroscopic motion upon tissue dehydration.^[^ ^37^ ^]^ The layers, in nearly perpendicular fibrillar directions, bring about a flat to helical shape change upon dehydration. Orthogonal layer shrinkage induces geometrical incompatibility, forming a saddle‐like configuration to accommodate elastic energy and the buildup of internal stresses within the layers.^[^ ^37^ ^]^ To minimize the energy, the seedpod halves twist helically away from each other, resulting in seed release.

Serotinous plants rely on the delayed release of mature seeds as a protection mechanism against extreme environmental conditions to optimize the success of seed dispersal and germination.^[^ ^7^, ^41^ ^]^ Such autonomous function requires energy management strategies and makes use of elastic energy storage for several years of time. In Banksia seedpods, known to survive bushfires, this has been addressed by multi‐step actuation occurring in response to different temperature and humidity conditions. First, the opening starts with the softening of the endocarp at increased temperatures (47–67 °C), which initiates the formation of a crack and an initial opening of the junction zone (Figure 3d, upper panel). This is attributed to the localized release of residual stresses generated by the shrinkage of the fiber bundles during follicle drying.^[^ ^7^ ^]^ Further opening for seed release requires wetting and drying cycles, which can be achieved during periods of rainfall. So, in the second step, the stored energy from the first steps (follicle drying and intensive heat during bushfire) activates the bending of the endo‐mesocarp bilayer and triggers the final release of the seeds.^[^ ^41^ ^]^

More generally, internally generated stresses play a prominent role in the mechanics and functionality of the tensional integrity (tensegrity) of biological systems, ranging from cells and cellular aggregations to skeletal elements such as bone‐muscle‐ligament systems.^[^ ^43^ ^]^ In these systems, tension‐bearing elements (flexible ligaments) and floating compressed components (rigid units) are mutually and hierarchically interconnected; the stored elastic energy in the tension‐bearing elements of the systems (e.g., actomyosin filaments in cells) shapes the overall geometrical arrangement to generate self‐equilibrium through force redistribution.^[^ ^44^ ^]^ Accordingly, modulation of internal forces and resultant strains can result in a new equilibrium state, changes in system geometry, and morphogenetic changes.^[^ ^45^ ^]^ Morphogenic changes can be cell‐driven in the form of myosin‐based contractions,^[^ ^46^ ^]^ cell division,^[^ ^47^ ^]^ biomechanical properties of cells,^[^ ^48^ ^]^ or Extracellular Matrix (ECM)‐driven, such as those in morphological tissue movement.^[^ ^49^, ^50^ ^]^ In embryonic chick development, the existence of stored ECM‐based elastic bending tension has been suggested as a driving source for folding the gut tube and external body wall.^[^ ^49^ ^]^ This large‐scale morphogenetic process has been hypothesized to be formed through a cycle of i) tension accumulation during embryo growth, ii) storage using ECM elasticity, and iii) dissipation by embryonic body folding.

### 2.3 Elastic Energy Storage for Rapid Movements

**Figure 4:**
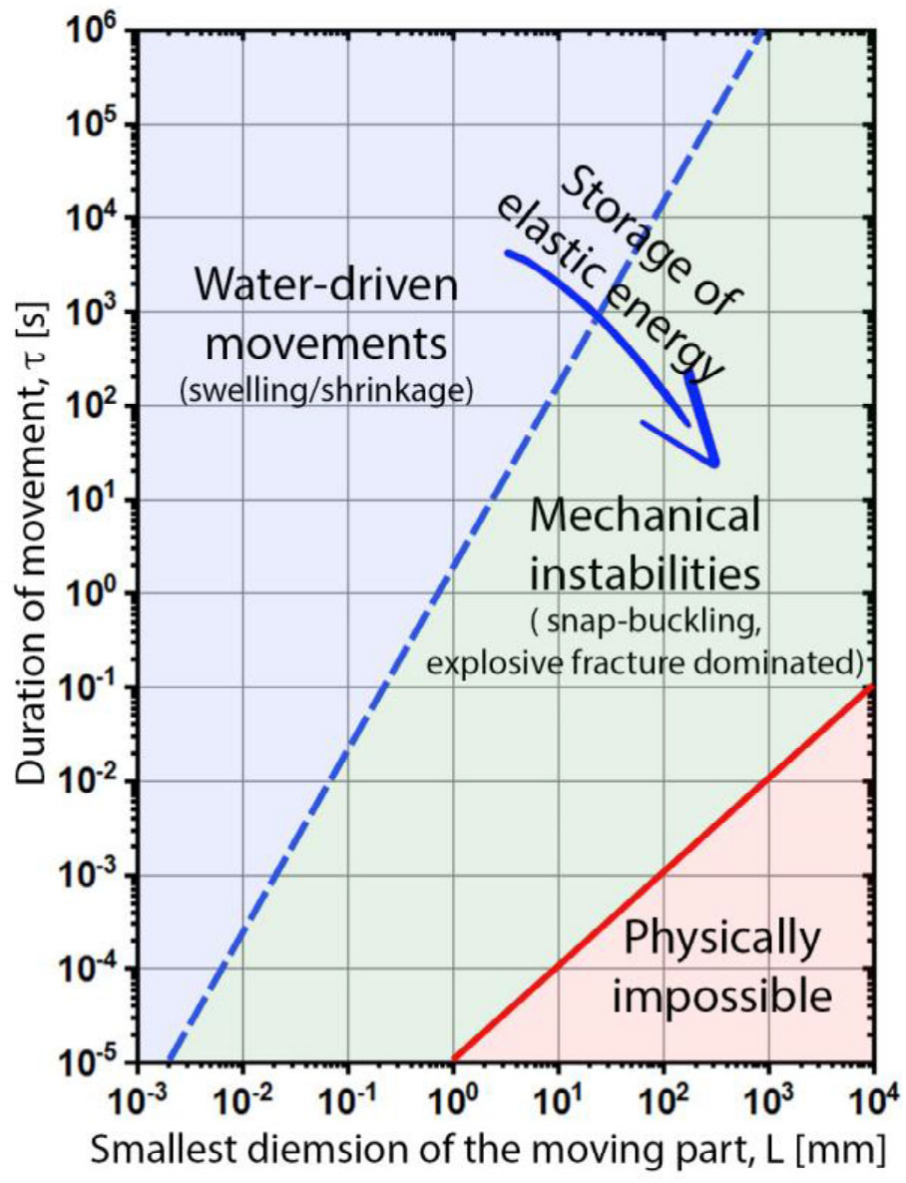
Physical classification of plant movement according to the duration of the movement as a function of the smallest dimension of the moving part. While plant actuation is limited to water movement and poroelastic time (blue dashed line), storing elastic energy combined with elastic instabilities can form biological springs, allowing ballistic movements. The plot is adapted with permission from^[^ ^52^ ^]^ Copyright 2005, AAAS.

The energy output from chemo‐mechanical conversion is not only limited by the actuation stress but sometimes also by the actuation strain rate, a limitation known as “Hill's law” for the case of muscle contraction, for example.^[^ ^51^ ^]^ This restriction arises because the actuation speed is governed by the rate of fluid transport^[^ ^52^ ^]^ and cross‐bridging dislocations by adenosine triphosphate (ATP).^[^ ^53^ ^]^ To overcome these speed limits, nature employs strategies to accumulate and amplify the kinetic energy by storing the energy in the form of elastic energy that can be released at a much faster rate than chemical energy conversion. In this way, movement can become i) more powerful, ii) faster, and iii) independent of the real‐time availability of the biofuel source (in the case of delayed actions). Such an energy management strategy allows biological systems to produce extremely rapid movements for functions such as prey capture, defense, locomotion, and reproduction.^[^ ^54^ ^]^ In plants, which are generally slow, the duration of the movement, τ, often exceeds the poroelastic timescale (τ_p_)–the time required for fluid to redistribute within the tissue following mechanical deformation. This poroelastic time is defined by τ_p_ = L^2^/D, where L is the transportation distance and D is the effective diffusion coefficient.^[^ ^39^, ^52^ ^]^ To exceed the poroelastic time limit dictated by water transportation, plants have combined water transport solutions with mechanical instabilities such as snap‐buckling, fracture, and cavitation^[^ ^39^, ^52^ ^]^ to store the elastic energies (**Figure** **4**). Release of this energy results in rapid movement at speeds comparable to those found in the animal kingdom.

One well‐known example is the *Venus flytrap* leaf, where the speed of snapping (≈100 ms) is a critical parameter for the functionality of the trap. This speed exceeds the limit of fluid flow‐based movements. While storage of elastic energy relies on an active biochemical component, the rapid closure of the lobes relies on a passive elastic component using snap‐buckling instability promoted by the geometrical constraint of the shell‐shaped doubly‐curved structures.^[^ ^9^ ^]^ Activating the trap‐snapping mechanism requires the accumulation of elastic energy so the lobes can bend outward (in an open position). Upon triggering the mechano‐sensitive hairs, actuated by generating an electrical signal, the accumulated stored energy buckles the lobes inside out and snaps the trap shut.^[^ ^39^, ^52^ ^]^ Observations of ballistic seed dispersal mechanisms have revealed striking strategies that allow for dispersion range expansion by kinematic amplification. In the genus *Impatiens*, a fracture release mechanism results in a launching velocity of 4 m ^−1^s^[^ ^55^ ^],^ resulting in a peak launch distance of 2 m^[^ ^56^ ^]^. The valves of the seedpod are composed of a bilayer structure, in which layers generate stress by water intake: the inner layer stretches, and the outer layer expands, restricted by geometrical confinement. This results in the formation of a strain mismatch and storage of elastic energy in the seedpod values. When a critical pressure is reached, splitting of the valves removes the geometrical confinement and releases the stored energy by shortening the inner layer and expanding the outer layer, together triggering a rapid (3 ms) coiling discharge of the seeds.^[^ ^56^ ^]^

**Figure 5:**
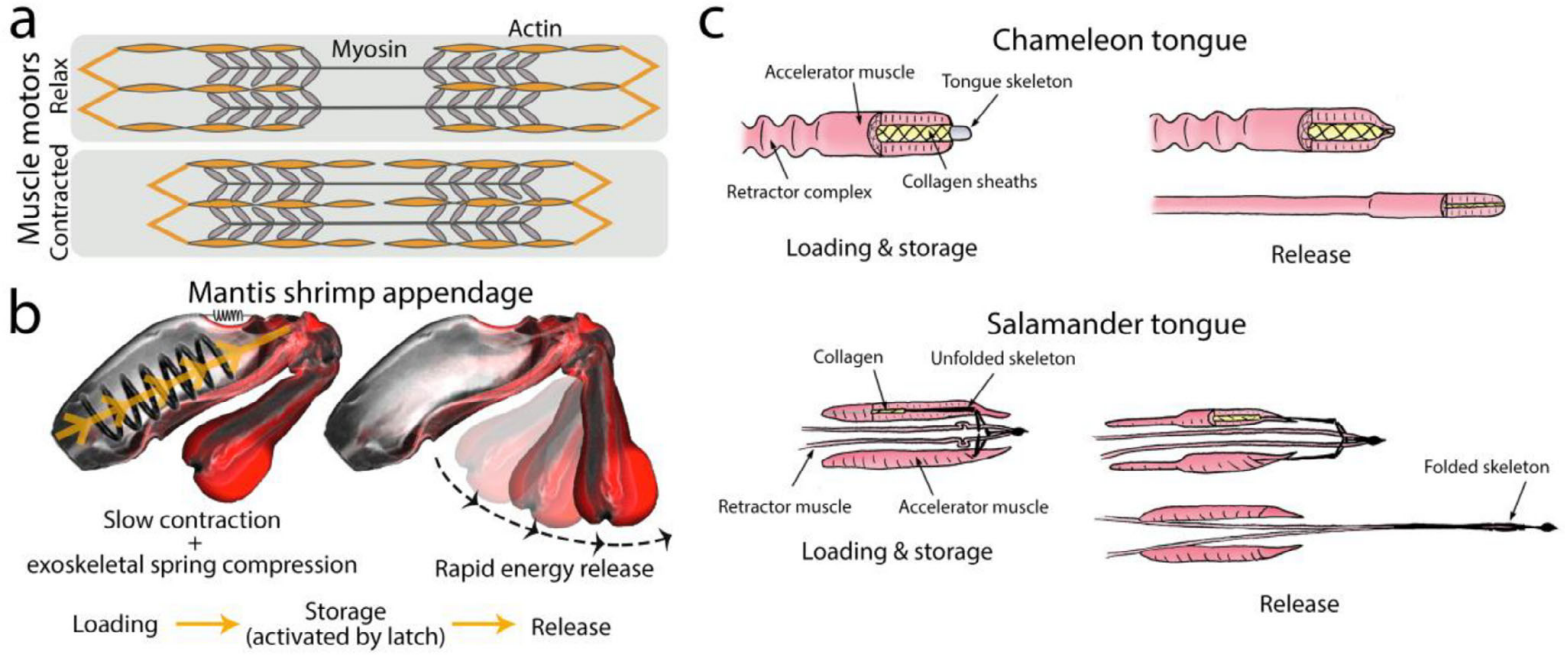
Examples of functions that rely on storing elastic energies generated by muscle motors. a) Muscle motors rely on chemical reactions; their actuation, based on sliding and contraction of actin‐myosin filaments, is very limited. Storage of elastic energy can exceed this limitation by delivering far greater forces at rapid speeds. b) Mantis shrimp appendage, which is powered by muscle motors, utilizes exoskeletal springs to change the lever and generate high‐energy smashes at high impact rates. The illustration is adapted with permission from^[^ ^59^ ^]^ Copyright 2018, Elsevier. c) The Ballistic movement of chameleon and salamander tongues relies on the storage of the elastic energy in collagen fibrils to far exceed the muscle contraction time. The drawings are adapted with permission from^[^ ^11^ ^]^ Copyright 2016, PLOS One.

Muscle tissue (**Figure** **5**a) is a universal driving motor of animal movement that follows a lever arm principle and can be optimized for speed or force (but not both simultaneously). This mainly arises from how the muscle motors work: long sarcomeres can generate contractions at a slow rate, and short sarcomeres produce fast contractions with low force.^[^ ^57^ ^]^ Nature has addressed this trade‐off by combining muscle motors, which power the movement, and spring‐linkage catapult systems, which amplify the power by accumulating elastic potential energy. Together, the system allows the rapid release of elastic energy and permits operations beyond the intrinsic muscle powers. Examples of such power amplifier systems can be found in mantis shrimp raptorial appendages (Figure 5b), known for their deadly strikes.^[^ ^5^ ^]^ In these appendages, the contraction of the extensor muscles in the merus results in the compression of an exoskeletal spring system embedded in the merus and saddle and the storage of elastic energy prior to the strikes.^[^ ^58^, ^59^ ^]^ This energy generated using flexor muscles activates the latches that secure energy storage; once the flexor muscle relaxes, the latches are released, and the stored elastic energy is delivered through a linkage mechanism connecting the springs to the striking segment, dactyl.^[^ ^5^ ^]^ This muscle‐spring combination provides an impressive system for delivering extremely rapid and forceful predatory strikes, which are not feasible using simple muscle contractions.

Similar energy management approaches have been widespread among arthropods. To overcome the temporal restrictions of muscle contractions, trap‐jaw ants rely on a muscle‐powered system modified by a latch‐lock mechanism. This combination makes it possible to load the mandible in the locked position slowly and then unlock the latch and rapidly release the stored elastic strain energy using closure muscles, resulting in the closure of mandibles at tremendous speeds.^[^ ^4^, ^60^ ^]^ In locusts^[^ ^61^, ^62^ ^]^ and fleas,^[^ ^63^ ^]^ distortion of the hard exoskeleton induced by slow muscle contraction can source the rapid release of elastic energy and trigger the propulsive movement of the hind legs.

Striking examples of ballistic tongue projection can be found in chameleons^[^ ^64^ ^]^ and salamanders,^[^ ^65^ ^]^ in which the tongues get all their impetus before launch at slow rates. In a chameleon, the energy generated by radial contraction of the accelerator muscle results in the deformation of the intralingual sheaths and a stretch of sheath collagen fibers, which stores the elastic energy.^[^ ^64^ ^]^ By sliding the collagen sheath over the tongue skeleton tip (Figure 5c), the block is overcome, and the tongue projection starts. Lungless salamanders similarly rely on stretching the collagen fibers to elastically load the sheath with strain energy.^[^ ^11^, ^66^ ^]^ However, in contrast to the chameleon, the lungless salamander's tongue skeleton accelerates off the muscles, and the folded skeleton projects toward the prey (Figure 5c).

## 3 Generation and Storage Mechanisms

**Figure 6:**
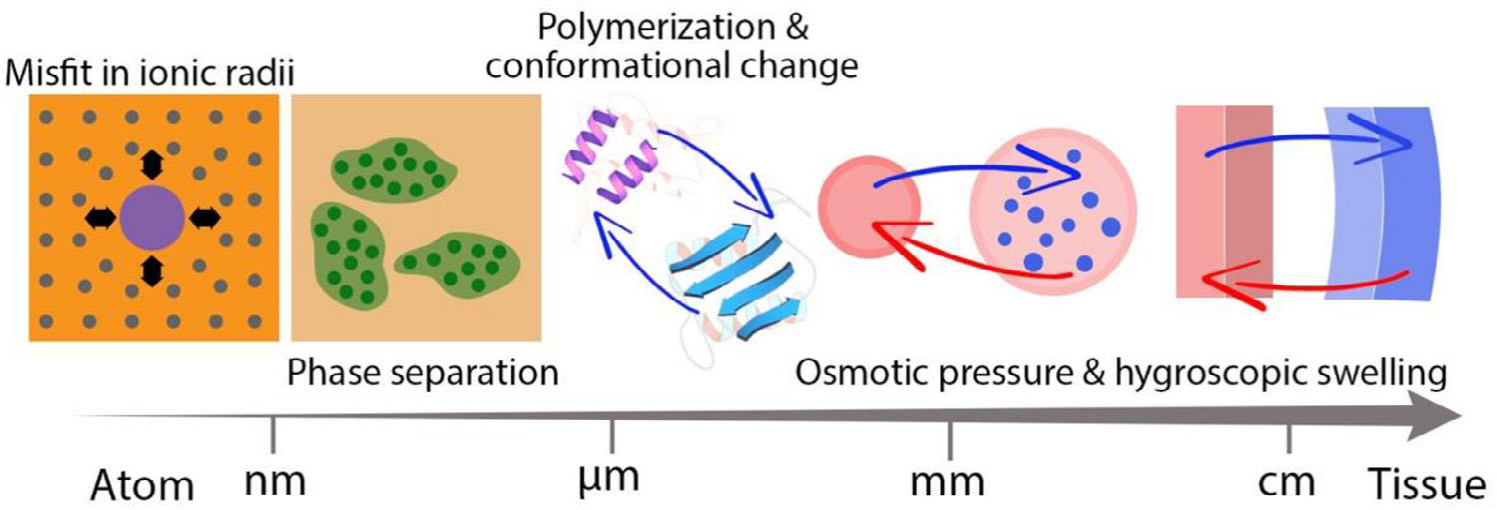
Generation of internal elastic energies can occur by volumetric changes across different length scales (atomic scales to tissue scales). See Sections "Crystallization with Atomic or Molecular Misfits"–"Osmotic Pressure and Hygroscopic Swelling".

Internal elastic energies result from volumetric changes that manifest themselves across multiple length scales (**Figure** **6**). In the biological world, volumetric changes can be generated during tissue formation and development, or later, due to swelling and shrinking in response to environmental stimuli such as temperature or humidity changes. As a consequence, internal stresses develop due to molecular size misfits at the nanoscale, as a result of phase transformations or osmotic pressure effects at the microscale or macroscopically, or from incompatibility in shrinkage and expansion. In engineering materials, plastic strains^[^ ^67^, ^68^ ^]^ and thermal expansion^[^ ^69^ ^]^ can induce volumetric changes and contribute to the formation of internal stresses. However, these effects are not typically associated with biological systems and are thus not discussed further here.

### 3.1 Crystallization with Atomic or Molecular Misfits

Misfits in ionic radii are well known to induce and store strain in various mineralized systems such as calcitic mollusk shells and human teeth. In calcite, Mg readily substitutes for Ca. Even though the thermodynamic solubility limit is only on the order of 1–2 at.%, many biominerals exhibit much higher Mg levels due to their formation via particles in an amorphous precursor.

When these nanoparticles are coherent, they exert compressive stresses on the matrix (as their average lattice parameter is smaller than that of the Mg‐poor matrix).^[^ ^25^ ^]^ These compressive stresses enhance the fracture toughness of the biomineral by resisting crack propagation. Homogeneous magnesium incorporation generally increases the hardness of calcite. Studies on synthetic single‐crystal calcite show a linear increase in hardness with increasing magnesium content, at least up to certain concentration limits. This increase in hardness is attributed to solid‐solution strengthening, where magnesium impurities hinder the movement of dislocations within the calcite lattice. The stress fields created by the size difference between Mg^2^⁺ and Ca^2^⁺ impede dislocation motion.^[^ ^25^, ^70^, ^71^, ^72^, ^73^ ^]^

When the Mg‐rich nanoparticles are not coherent with the matrix, it has been shown that, despite the lack of compressive stresses, they exhibit a precipitation hardening mechanism, increasing the hardness due to interactions between nanoparticles and dislocations. This has been shown to significantly enhance the hardness beyond that achieved by solid‐solution hardening alone. Calculations suggest that precipitation hardening can lead to at least a twofold increase in hardening compared to a homogenous Mg distribution. Other than the formation of high‐Mg nanoparticles, the distribution of magnesium within biominerals can be heterogeneous in other ways. For instance, in sea urchin teeth, the polycrystalline matrix has a much higher Mg content compared to the single‐crystal needles and plates.^[^ ^70^, ^74^ ^]^ Furthermore, Mg concentration gradients can exist within individual crystal elements. As the next hierarchical level of inhomogeneous Mg distribution, it has been shown that the brittle star not only exhibits coherent Mg‐rich nanoprecipitates but also demonstrates layers with varying nanoparticle concentrations.^[^ ^8^, ^25^ ^]^ This allows not only to have the entire matrix under compression but also to have alternating levels of compressive stresses, which serve as an additional crack‐deflecting mechanism.

Similar to what was observed for Mg in biogenic calcite, it was shown in human tooth enamel that the presence of chemical gradients at multiple hierarchical levels, particularly within individual hydroxylapatite (OHAp) crystallites and potentially across the rod/interrod microstructure, plays a crucial role in modulating its properties.^[^ ^75^, ^76^ ^]^ These gradients involve variations in minority ion concentrations such as magnesium (Mg^2^⁺), sodium (Na⁺), fluoride (F−), and carbonate (CO_3_^2−^) substituting within the OHAp lattice. Due to the differing ionic radii compared to calcium (Ca^2^⁺), these substitutions lead to variations in lattice parameters (specifically, the “a” and “c” axes) of the OHAp structure. Synchrotron X‐ray microdiffraction studies have shown that these lattice parameter differences are correlated with the rod/interrod microstructure. Atom probe tomography further reveals a core‐shell structure within crystallites, where the core is enriched in Na, F, and C, often flanked by Mg‐rich layers, while the shell has lower impurity concentrations. These lattice parameter variations, resulting from compositional gradients, effectively induce eigenstrains within the crystallites, generating net residual compressive stress. Finite‐element modeling predicts that the core regions experience net tensile stress, particularly in the Mg‐rich layers, while the crystallite shell is under compressive residual stresses. These residual stresses have significant functional consequences: the tensile stress in the core is predicted to increase its solubility, which aligns with observations of preferential dissolution and etching of the core in acidic conditions, a factor relevant to tooth decay. Conversely, the compressive stresses in the crystallite shell may enhance mechanical resilience by helping to impede crack initiation and deflect cracks, thereby increasing the enamel's tolerance to mechanical stress.

### 3.2 Crystallization with Phase Separation

It has been shown that in some biominerals that exhibit higher magnesium content (above 14 mol.%), high‐Mg calcite nanoparticles can form within a lower‐Mg calcite matrix (inhomogeneous Mg distribution).^[^ ^77^, ^78^ ^]^ This nanostructure, believed to be formed through the spinodal decomposition of an amorphous precursor, is a widespread biostrategy to enhance mechanical durability. There are two scenarios in this latter case. The first is that the Mg‐rich nanoparticles are coherent with the lattice (as mentioned above), while the second scenario is when such nanoparticles are not coherent with the matrix.^[^ ^78^ ^]^

### 3.3 Polymerization and Conformational Change

The generation and storage of internal forces can occur during polymerization and conformation changes in polymeric structures. The nonequilibrium polymerization of actin filaments (F‐actin) generates forces that are key to cell and tissue stiffness, motility, and morphogenesis.^[^ ^79^ ^]^ Through this protein polymerization, chemical energy, which can be stored in cells, converts to mechanical force. The chemo‐mechanical conversion can be either with myosin hydrolyzing ATP, as in skeletal muscle contraction, or using actin polymerization motor proteins, which rely on the assembly of monomeric actin molecules (G‐actin).^[^ ^80^ ^]^ Likewise, microtubule polymerization and depolymerization can generate pushing and pulling forces by assembling and disassembling αβ‐tubulin dimers. The forces generated by the shortening or lengthening of the microtubules are responsible for intracellular motility, such as the positioning of chromosomes, mitotic spindles, and nuclei in cells.^[^ ^81^ ^]^ Obviously, in the direct transformation of generated mechanical forces to kinematic energy, the mechanical energy will be consumed, and there will be no energy storage. Yet, it has been shown that intracellular forces can be partially stored through conformational changes such as nonequilibrium transition between α‐helices and β‐sheets of vimentin intermediate filaments.^[^ ^82^ ^]^ Moreover, analogous to the phase transition between the low‐strain and high‐strain phases of pseudoelastic shape memory alloys, biological materials can take advantage of reversible conformational changes of proteins to store elastic energies. Examples include the egg capsules of welk in which, upon stretching, the α‐helix protein arrangement (low‐strain phase) can be transformed to the β* arrangement (high‐strain phase).^[^ ^83^ ^]^ Remarkably, during the relaxation and despite the partial energy dissipation, there is a complete recovery to the α‐phase.

### 3.4 Osmotic Pressure and Hygroscopic Swelling

Water is incompressible and cannot be used for elastic energy storage. However, it is a great medium for transmitting hydrostatic pressure so that water uptake into a cell, whether in plants or animals, can lead to its expansion and intracellular hydrostatic pressure. Limiting the water‐induced cell expansion by rigid containers, such as stiff cell walls in plants, generates tensile stresses in the container walls. So, anisotropies in cell wall rigidity, size, and density can be source mismatches in induced stresses and can be used for generating and storing elastic energies (Figure 3). Therefore, by actively controlling solutes and regulating osmotic pressure, plants and soft tissues can gain mechanical stability to promote growth or shape‐morphing actuation in a reversible manner.^[^ ^84^, ^85^ ^]^ On the other hand, nonuniform osmotic‐driven swelling in the tissue, such as cartilage, can form non‐uniform internal strain fields. This has been attributed to the heterogeneities in density and distribution of negatively charged proteoglycans embedded in collagenous networks.^[^ ^86^, ^87^ ^]^

In addition to intracellular‐driven osmosis, hygroscopic swelling can contribute to the formation of internal elastic energies. This is particularly important in plant materials where humidity conditions can drastically change in the environment. Hygromorphic materials such as wood can undergo dimensional changes in response to moisture level changes. The orientation of cellulose fibers in the wood cell walls converts isotropic swelling to directional movements.^[^ ^88^ ^]^ Therefore, besides the mismatches between the hygroscopic characteristics of fibers and matrices, parameters such as gradients in hydration/ dehydration and/or the presence of anisotropic wall structure can lead to mismatches in mechanical deformations and formation of internal stresses, which can drive many passive movements in plant cells.^[^ ^89^ ^]^

## 4 Measurement of Internal Strains: Challenges and Approaches

Measuring internal strains in biological materials often comes with three main challenges: i) quantifying strain distribution and limits to imaging resolution, ii) methodological sensitivity, and iii) strain deviation from the native condition, as briefly introduced in the following.

### 4.1 Strain Distribution and Imaging Resolution

The embodied structural complexities of biological materials lead to location‐specific variations in their mechanical characteristics. Investigating internal strains is no exception; therefore, studies often require characterization approaches such as mapping, which offer pathways for identifying localized variations at different material hierarchical levels.

### 4.2 Methodological Sensitivity

Since mechanical stresses cannot be measured directly, internal strain quantification often relies on measuring changes in physical dimensions – deformations. Mechanical deformations on macro or micro scales can be measured using conventional microscopic methods, such as digital image correlation. However, measuring internal strains may require methodological approaches capable of resolving changes in crystal lattice spacing, length of chemical bonds, and size and spacing of ultrastructural features such as fibrillar spacing, pore size, etc.

### 4.3 Strain Deviation

Compared to their engineering counterparts, internal strains in biological materials can rapidly change with environmental factors (e.g., changes in humidity, temperature, and sample preparation effects). So, they come with the complexity of needing to ensure that they are probed in their native conditions. Accordingly, besides the considerations for the storage of biological materials, special attention should be paid to sample preparation and exposure conditions during the measurements (see *Time‐radiation damage effects*)

Accordingly, despite the fact that over the last decades, several techniques have been developed to evaluate internal strains/stresses, the experimental assessment of the localized stresses in biological materials remains quite challenging. In the following, we introduce the main approaches that have been employed for investigating the mapping the internal strains in biological models:

### 4.4 High‐Resolution X‐ray Diffraction Microscopy

Microfocus X‐ray diffraction is a potent technique for real‐time strain/stress measurements with micron‐size probing resolution.^[^ ^90^ ^]^ The technique is used to measure the local elastic strain, using the Bragg relation that directly links the positions of diffraction peaks to the lattice parameters (d‐spacing) of a crystalline structure. However, to provide sufficient X‐ray flux into the submicron gauge volumes, the technique had to be conducted in advanced (high‐energy) synchrotron beamlines. Similar to electron diffraction techniques, microfocus X‐ray diffraction comes with its own drawbacks for sample preparation with restricted sizes, in which the free surfaces of the sample can induce or relax the residual stress/strain in the sample. This method has been extensively used for characterizing the internal strains in plants,^[^ ^91^, ^92^ ^]^ mineralized collagen,^[^ ^26^, ^30^ ^]^ and highly mineralized skeletons.^[^ ^93^ ^]^ In highly mineralized tissues where the matrices are ceramics, reasonably high stresses are obtained by even low lattice strains (due to high elastic modulus). X‐ray diffraction methods allow for reliable measurement of very small lattice strains, even as low as 0.001%. The combination of highly monochromatized x‐ray beams, combined by analyzer crystals preceding the detectors, is what allows for such reliable low detection limits. In addition, such beamlines offer the ability to perform in‐situ heating, gas flow, and mechanical testing.^[^ ^94^ ^]^

### 4.5 Time‐Radiation Damage Effects

Radiation damage resulting from high X‐ray beam flux is a major limitation in characterizing biological tissues.^[^ ^95^, ^96^ ^]^ On the other hand, inducing X‐ray beam radiation damages and measuring the material conformational changes by releasing the stored energies has been a recent approach for detecting and quantifying the stored elastic strains in biomineralized tissues.^[^ ^97^ ^]^ With this method, effects such as collagen degradation and strain relaxation at mineral crystals can be measured as a function of exposure time and radiation dose.^[^ ^97^ ^]^

### 4.6 Confocal Raman Microscopy

Raman spectroscopy provides a powerful and non‐destructive route to determining elastic strains in Raman‐active solids. This method, which relies upon the inelastic scattering of photons, elastic deformations, and the corresponding changes in chemical bond lengths, can be accurately determined through changes in the vibration frequency of the molecules.^[^ ^98^ ^]^ These changes can be recorded by the peak shifts in associated bands in Raman spectra. So, in contrast with X‐ray diffraction microscopy, the method provides an indirect way for quantifying the material deformations,^[^ ^99^ ^]^ and it has been extensively used for characterizing the distribution of residual strains in biomineralized tissues.^[^ ^100^, ^101^, ^102^ ^]^ Depending on the peak intensity and magnitude of the strains, the peak shift can be large or stay below one Raman shift,^[^ ^103^ ^]^ so spectrometers with high spectral resolution are required for tracing minor strains. The technique requires minimal sample preparation and can map residual strains with high resolution and 3D, so it has been used to investigate internal strain fields.^[^ ^101^ ^]^ Recent developments in the method have allowed investigation of the evolution of anisotropic elastic strain fields in crystalline solids in the presence of localized applied forces in operando, 3D, and with sub‐micron resolution.^[^ ^103^ ^]^

## 5 Outlook

**Figure 7:**
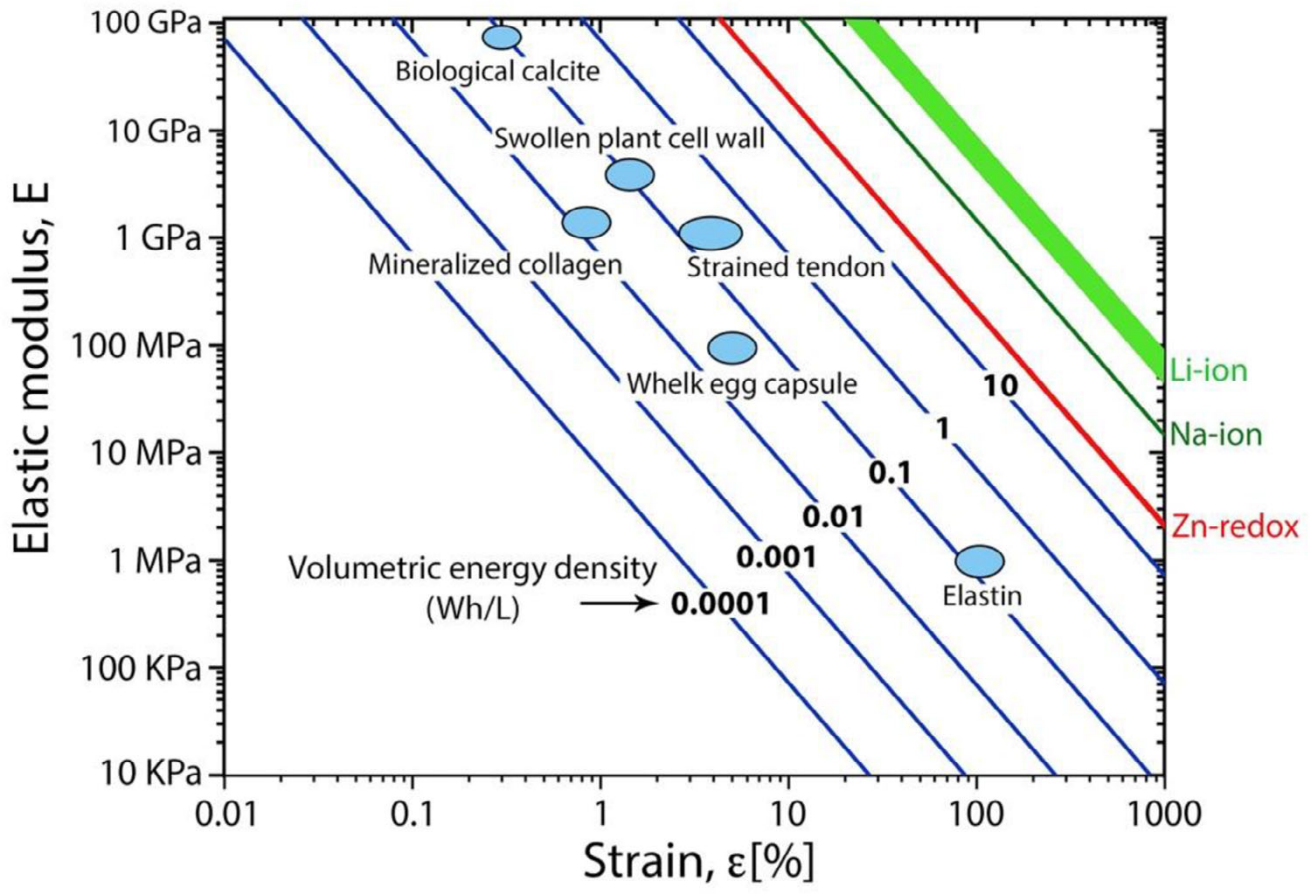
Stored energy in biological materials. The volumetric energy density increases linearly with modulus and quadratically with strain (Equation (1)). Blue lines indicate equal values of volumetric energy density. For comparison, the typical energy density values for electrical batteries^[^ ^104^ ^]^ are also indicated at the position where modulus and strain in an elastic material would correspond to the same level of stored energy. Several examples from biological materials are indicated in the graph: Organic inclusions in calcite,^[^ ^25^ ^]^ plant cell wall swelling,^[^ ^105^ ^]^ collagen mineralization in bone,^[^ ^26^, ^27^ ^]^ tendon under tension, whelk egg capsule^[^ ^83^, ^106^ ^]^ and elastin.^[^ ^107^ ^]^

Internal stresses are a widely underestimated strategy for storing energy in biological and bio‐inspired materials. The elastic energy density stored in any material is estimated with the following equation:

(1) $$W=\frac{1}{2}E\varepsilon^{2}$$

where *E* and ɛ are the elastic modulus and strain, respectively. Of course, for more complex strains, this expression is a tensor product, but for the order of magnitude estimates we will be using here, this gives a first estimation. **Figure** **7** shows lines with equal values of *W* together with values of elastic energy storage capacity of various materials discussed in this review. It turns out that many of these materials are able to reach a volumetric energy density in the order of 0.1 Wh L^−1^, despite widely different strains and elastic moduli. For comparison, the energy density in electrical batteries (that is, between one to three orders of magnitude larger) is also indicated in Figure 7.

One of the big advantages of elastic energy storage is the speed at which the energy can be released, which is advantageous when large strain rates are needed, e.g., for jumping. Indeed, chemical energy conversion (as occurring in muscle contraction) is limited by reaction rates that are comparatively limited, and intermediate elastic energy storage in tendons or other elastic materials is advantageous.^[^ ^108^, ^109^ ^]^ This is also visible in plant movements, as described in Figure 4. Another advantage is that no additional device, such as a battery, is needed for energy storage since the structural components of the device serve for the elastic energy storage.

While energy storage is considered one of the most pressing areas of technological development, hardly any research addresses elastic energy storage based on internal strains. Of course, the volumetric energy density is significantly lower than electrochemical batteries (Figure 7) and much lower than that of fossil fuels, yet it still has several interesting advantages. First, it can be released at very high rates without a heat burst that would typically accompany an explosive chemical energy conversion. This property is used by biological organisms for jumping, throwing, and hammering. It also does not require specific containers, but – as the examples of biological materials show – the whole body can in principle be used as an “elastic battery”.

In the engineering sciences, residual internal stresses are widely considered in technological applications,^[^ ^12^, ^13^ ^]^ from arrested springs to stress design in railway tracks^[^ ^110^ ^]^ or reinforced concrete.^[^ ^111^ ^]^ However, this is rarely done with the perspective of energy storage, which implies temporary internal stresses that can be released on demand. Further research will be required to develop and investigate elastic energy storage systems that go well beyond a mousetrap that really just works, because the elastic energy of the spring is released at a rate that outcompetes the speed at which the mouse can actuate its muscles. The research opportunity is to develop sustainable designs where any material parts (and not just dedicated springs) may be considered for elastic energy storage, thereby making a multifunctional use of structural components of any device. Natural materials, as described in this review, may serve as blueprints for such approaches.

## Conflict of Interest

The authors declare no conflict of interest.
